# Crystal structure of tetra­kis­(μ-2-hy­droxy-3,5-di­isoprop­yl­benzoato)bis­[(dimethyl sulfoxide)copper(II)]

**DOI:** 10.1107/S205698902400166X

**Published:** 2024-02-27

**Authors:** Daniel G. Shlian, Rachael H. Summers, Katelyn Martinez, Rita K. Upmacis

**Affiliations:** a Department of Chemistry, Columbia University, New York, NY 10027, USA; b Department of Chemistry & Physical Sciences, Pace University, New York, NY 10038, USA; University of Missouri-Columbia, USA

**Keywords:** crystal structure, binuclear copper, 3,5-diiso­propyl­salicylate, dimethyl sulfoxide.

## Abstract

Metal complexes of 3,5-diiso­propyl­salicylate are reported to have anti-inflammatory and anti-convulsant activities. The title binuclear copper complex, [Cu_2_(C_13_H_17_O_3_)_4_(C_2_H_6_OS)_2_] or [Cu(II)_2_(3,5-DIPS)_4_(DMSO)_2_], contains two five-coordinate copper atoms that are bridged by four 3,5-diiso­propyl­salicylate ligands and capped by two axial dimethyl sulfoxide (DMSO) moieties. Each copper atom is attached to four oxygen atoms in an almost square-planar fashion, with the addition of a DMSO ligand in an apical position leading to a square-pyramidal arrangement. The hy­droxy group of the diiso­propyl­salicylate ligands participates in intra­molecular O—H⋯O hydrogen-bonding inter­actions.

## Abstract 2

A solution of 2-hy­droxy-3,5-bis­(1-methyl­eth­yl)benzoic acid copper(II) hydrate (C_26_H_34_CuO_6_·*x*H_2_O), also known as copper(II) 3,5-diiso­propyl­salicylate hydrate, in dimethyl sulfoxide (DMSO) affords crystals of tetra­kis-3,5-diiso­propyl­salicylatobis-di­methyl­sulfoxido­dicopper(II), [Cu(II)_2_(3,5-DIPS)_4_(DMSO)_2_], upon evaporation. The structure has an empirical formula of [Cu_2_(C_13_H_17_O_3_)_4_(C_2_H_6_OS)_2_] and consists of a centrosymmetric binuclear copper complex surrounded by four 3,5-diiso­propyl­salicylate ligands. Each copper atom is attached to four oxygen atoms in an almost square-planar fashion, with the addition of a DMSO ligand in an apical position leading to a square-pyramidal arrangement.

## 1. Chemical context

A variety of binuclear Cu^II^ complexes bound to carboxyl­ate moieties and donor ligands are known (Doedens, 1976 ▸). These include, for instance, Cu^II^ complexes with di­alkyl­salicylates (Morgant *et al.*, 2000 ▸; Benisvy *et al.*, 2006 ▸; Seguin *et al.*, 2021 ▸) and non-steroidal anti-inflammatory drugs (NSAIDs) (Dendrinou-Samara *et al.*, 1990 ▸; Kovala-Demertzi *et al.*, 1997 ▸; Guessous *et al.*, 1998 ▸; Greenaway *et al.*, 1999 ▸; Viossat *et al.*, 2003 ▸, 2005 ▸). With regard to Cu^II^ complexes with di­alkyl­salicylates, several complexes containing 3,5-diiso­propyl­salicylate (3,5-DIPS) of the type [Cu(II)_2_(3,5-DIPS)_4_(*L*)_2_], in which *L* is a donor mol­ecule, are known and have been characterized by electron paramagnetic resonance (EPR), infrared (IR) and ultraviolet–visible (UV–Vis) spectroscopies (Greenaway *et al.*, 1988 ▸). However, compounds featuring dimethyl formamide (DMF) and di­ethyl­ether giving rise to [Cu(II)_2_(3,5-DIPS)_4_(DMF)_2_] and [Cu(II)_2_(3,5-DIPS)_4_(OEt_2_)_2_], respectively, have been characterized by X-ray diffraction (Morgant *et al.*, 2000 ▸).

In contrast to the binuclear structures of these copper compounds, the structure of the zinc counterpart that is obtained from dimethyl sulfoxide (DMSO) is mononuclear, [Zn(II)(3,5-DIPS)_2_(DMSO)_2_], as determined by X-ray crystallography (Morgant *et al.*, 1998 ▸). Since Cu^II^ and Zn^II^ complexes of 3,5-DIPS are of inter­est because they inhibit polymorphonuclear leukocyte oxidative metabolism *in vitro* and have anti­convulsant activity (Morgant *et al.*, 1998 ▸, 2000 ▸), it is pertinent to determine the structure of the corresponding copper complex. Therefore, herein, we describe the X-ray crystallography structure of the binuclear copper complex, [Cu(II)_2_(3,5-DIPS)_4_(DMSO)_2_], which is obtained from a solution of copper(II) 3,5-diiso­propyl­salicylate hydrate in DMSO. 
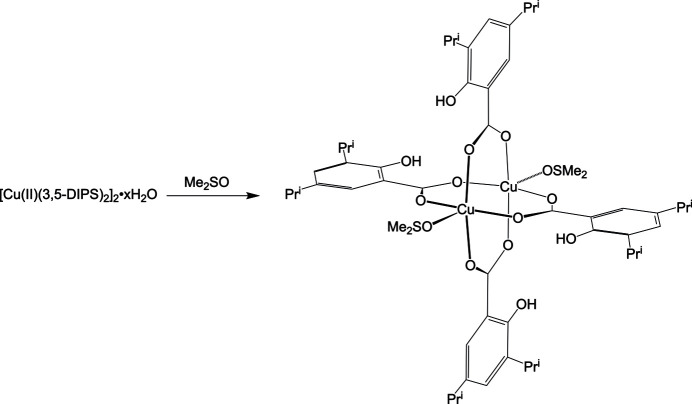


## 2. Structural commentary

**Figure 1:**
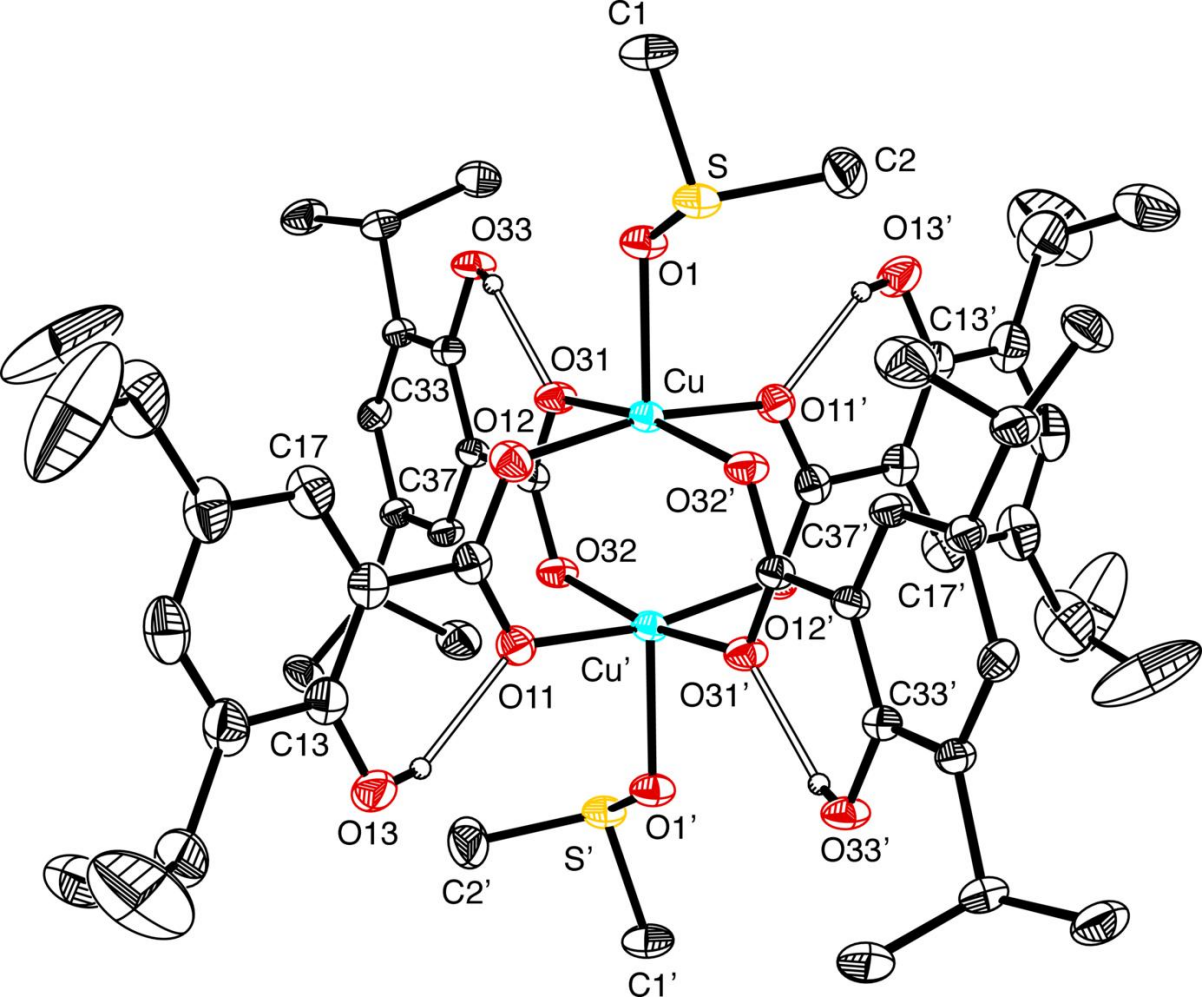
Crystal structure of [Cu(II)_2_(3,5-DIPS)_4_(DMSO)_2_]. For clarity, hydrogen atoms on carbon have been omitted. The OH group is disordered over two sites on each aromatic ring, namely C13/C17 and C33/C37, with site occupancy ratios of 0.723 (6):0.277 (6) and 0.859 (5):0.141 (5), respectively; for clarity, only the major component with its hydrogen-bonding inter­actions is illustrated. Displacement ellipsoids are shown at the 30% probability level.

The structure of [Cu(II)_2_(3,5-DIPS)_4_(DMSO)_2_], shown in Fig. 1 ▸, reveals that the compound is a centrosymmetric binuclear complex containing two copper atoms, with a Cu⋯Cu distance of 2.6170 (7) Å, that are bridged by four 3,5-diiso­propyl­salicylate (DIPS) ligands. The inter­nal symmetry element (inversion center) allows for half of the complex to be represented in the asymmetric unit. As found with other [Cu(II)(3,5-DIPS)] compounds, the OH moiety attached to the aromatic ring is not involved in bonding to the copper centers (Ranford *et al.*, 1993 ▸; Morgant *et al.*, 2000 ▸). Each Cu atom forms an almost square-planar geometry with four oxygen atoms from the carboxyl­ate groups of the 3,5-DIPS moieties, with Cu—O distances ranging between 1.958 (2) and 1.972 (2) Å. The O—Cu—O angles range from 88.12 (9) to 90.21 (9)° for *cis* and 168.77 (7) to 168.80 (8)° for *trans* positions, indicating that the arrangement is close to an idealized square-planar geometry.

Each Cu atom is also capped by a DMSO ligand in the apical position with a Cu—OSMe_2_ distance of 2.1226 (19) Å leading to a square-pyramidal arrangement. The O11—Cu—OSMe_2_, O12—Cu—OSMe_2_, O31—Cu—OSMe_2_ and O32—Cu—OSMe_2_ angles range from 95.41 (8) to 95.79 (7)°, indicating a slight deviation from the 90° angle expected for an idealized square-pyramidal arrangement. In accord with this description, the τ_5_ geometry index (Addison *et al.*, 1984 ▸) for the [CuO_5_] moiety is close to zero (0.00005); for reference, a τ_5_ geometry index of 0.00 corresponds to a square-pyramidal geometry while a value of 1.00 corresponds to an idealized trigonal–bipyramidal geometry (Addison *et al.*, 1984 ▸; Palmer & Parkin, 2014 ▸).

The OH group is disordered over two sites on each aromatic ring, namely C13/C17 and C33/C37, with site occupancy ratios of 0.723 (6):0.277 (6) and 0.859 (5):0.141 (5), respectively. This type of disorder has previously been observed for other [Cu(II)(3,5-DIPS)] compounds, such as [Cu(II)_2_(3,5-DIPS)_4_(DMF)_2_] and mononuclear [Cu(II)(3,5-DIPS)_2_(1,10-phenanthroline)] (Morgant *et al.*, 2000 ▸; Ranford *et al.*, 1993 ▸). For comparison, the OH group disorder for [Cu(II)_2_(3,5-DIPS)_4_(DMF)_2_] occurs in a 64:36 ratio for each 3,5-DIPS ligand (Morgant *et al.*, 2000 ▸), while for the mononuclear Cu structure containing 1,10-phenanthroline, the disorder occurs in a 60:40 ratio (Ranford *et al.*, 1993 ▸).

## 3. Supra­molecular features

**Figure 2:**
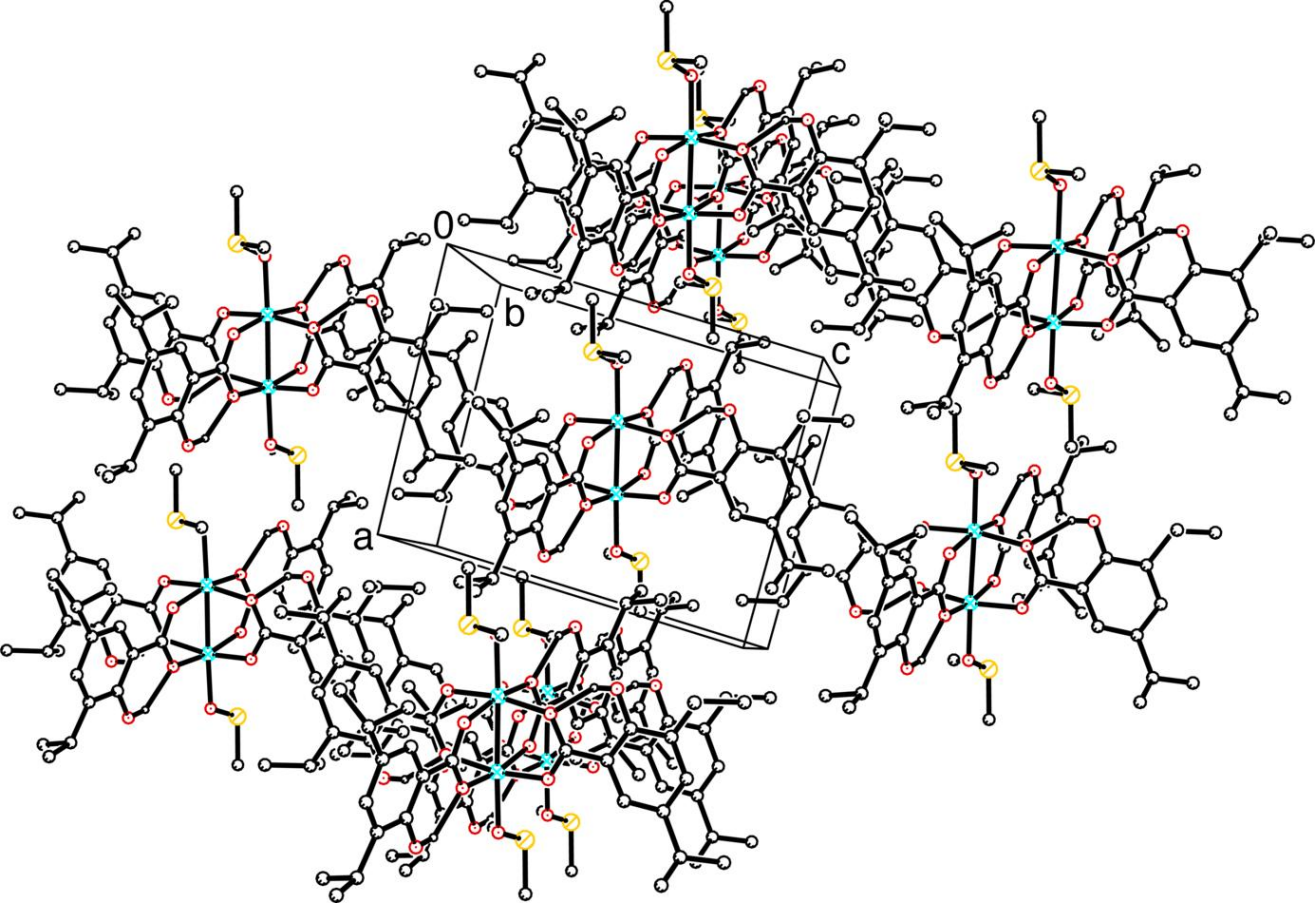
Unit-cell packing diagram of [Cu(II)_2_(3,5-DIPS)_4_(DMSO)_2_].

Fig. 2 ▸ shows the packing in the unit cell. There are no significant inter­molecular inter­actions. However, the structure displays hydrogen-bonding inter­actions within the mol­ecule, which are those between the aromatic OH groups and an oxygen atom of the carboxyl­ate group within the 3,5-DIPS ligand. The hydrogen bond O—H⋯O distances and angles for O13—H⋯O11, O13*A*—H⋯O12, O33—H⋯O31 and O33*A*—H⋯O32 are reported in Table 1 ▸. As a result of the OH disorder observed, there are two O—H⋯O distances recorded for each aromatic ring.

The intra­molecular hydrogen-bond distances and angles reported in Table 1 ▸ are within the typical range of other reported [Cu(II)_2_(3,5-DIPS)_4_(*L*)_2_] compounds. For instance, the hydrogen-bond O—H⋯O distances and angles reported for [Cu(II)_2_(3,5-DIPS)_4_(DMF)_2_] range from 2.493 (4) to 2.559 (4) Å and 137.0 to 147.6°, respectively (Morgant *et al.*, 2000 ▸).

Similar intra­molecular hydrogen-bond O—H⋯O distances and angles are also reported for related binuclear copper(II) compounds containing 3,5-diiso­butyl­salicylate (3,5-DIBS) that also bear a ring mol­ecule with *ortho* carb­oxy­lic and alcohol functional groups. For example, [Cu(II)_2_(3,5-DIBS)_4_(CH_3_OH)_2_] displays intra­molecular hydrogen-bond O—H⋯O distances ranging from 2.575 (6) to 2.565 (6) Å, and O—H⋯O angles between 131 and 146° (Benisvy *et al.*, 2006 ▸).

## 4. Database survey

Crystal structures of 3,5-diiso­propyl­salicylate copper(II) complexes bound to additional axial donors (*L*) of the form [Cu(II)_2_(3,5-DIPS)_4_(*L*)_2_] are known, and include the DMF and di­ethyl­ether ligated compounds (Morgant *et al.*, 2000 ▸). The title compound, as well as others containing different solvent mol­ecules, such as the di­aqua variant, have been previously characterized as [Cu(II)_2_(3,5-DIPS)_4_(*L*)_2_] compounds, but crystal structures were not published (Greenaway *et al.*, 1988 ▸; Ranford *et al.*, 1993 ▸).

Other ternary Cu^II^ complexes containing solvents bound in the axial positions where 3,5-DIPS is replaced by non-steroidal anti-inflammatory drugs (NSAIDs) of the form [Cu(II)_2_(NSAID)_4_(*L*)_2_] are also known. These include: [Cu(II)_2_(naproxen)_4_(DMSO)_2_] (Dendrinou-Samara *et al.*, 1990 ▸); [Cu(II)_2_(diclofenac)_4_(DMF)_2_] (Kovala-Demertzi *et al.*, 1997 ▸); [Cu(II)_2_(indomethacinate)_4_(DMF)_2_] (Guessous *et al.*, 1998 ▸); [Cu(II)_2_(niflumate)_4_(DMSO)_2_] (Greenaway *et al.*, 1999 ▸); [Cu(II)_2_(aspirinate)_4_(DMSO)_2_] (Viossat *et al.*, 2003 ▸) and [Cu(II)_2_(niflumate)_4_(H_2_O)_2_·4DMA] (DMA = di­methyl­acetamide; Viossat *et al.*, 2005 ▸). Notably, the title compound has similar structural features to previously characterized NSAID analogs (Table 2 ▸).

Related ternary binuclear copper(II) containing 3,5-diiso­butyl­salicylate (3,5-DIBS) compounds that contain solvent ligands are also known. For instance, compounds such as [Cu(II)_2_(3,5-DIBS)_4_(CH_3_OH)_2_] and [Cu(II)_2_(3,5-DIBS)_4_(EtOH)_2_] have also been characterized (Benisvy *et al.*, 2006 ▸; Seguin *et al.*, 2021 ▸).

In contrast to these binuclear copper structures, other motifs are observed for different metals. For example, the zinc compound contains a mononuclear zinc center surrounded by two 3,5-DIPS ligands and two DMSO solvent mol­ecules of the form [Zn(II)(3,5-DIPS)_2_(DMSO)_2_] (Morgant *et al.*, 1998 ▸). The Zn^II^ complex of 3,5-DIPS has anti­convulsant activity and inhibits polymorphonuclear leukocyte oxidative bursts *in vitro* (Morgant *et al.*, 1998 ▸). The (3,5-DIPS) compounds of Fe and Mn also exhibit anti-oxidant activity (Tavadyan *et al.*, 2004 ▸).

## 5. Synthesis and crystallization

A green block of [Cu(II)_2_(3,5-DIPS)_4_(DMSO)_2_] suitable for X-ray diffraction was obtained by directing a flow of air above a solution of copper(II) 3,5-diiso­propyl­salicylate hydrate (0.07 g, 0.14 mmol) in DMSO (15 mL) over several days at room temperature. In the absence of a flow of air, crystals were also obtained over a period of 11 months.

## 6. Refinement

Crystal data, data collection and structure refinement details are summarized in Table 3 ▸. Disordered groups were treated using fully constrained refinement (site occupancies, coord­inates, thermal parameters) with *SHELXTL* (Version 2014/7; Sheldrick, 2008 ▸). Hydrogen atoms on carbon were placed in calculated positions (C—H = 0.95–1.00 Å) and included as riding contributions with isotropic displacement parameters *U*_iso_(H) = 1.2*U*_eq_(C*sp* ^2^) or 1.5*U*_eq_(C*sp* ^3^). The disorder of the hydroxyl groups was modeled such that the sum of their site occupancies is 1.0.

## Supplementary Material

Crystal structure: contains datablock(s) I. DOI: 10.1107/S205698902400166X/ev2003sup1.cif

Structure factors: contains datablock(s) I. DOI: 10.1107/S205698902400166X/ev2003Isup2.hkl

CCDC reference: 2333981

Additional supporting information:  crystallographic information; 3D view; checkCIF report

## Figures and Tables

**Table 1:** Hydrogen-bond geometry (Å, °)

*D*—H⋯*A*	*D*—H	H⋯*A*	*D*⋯*A*	*D*—H⋯*A*
O13—H13*B*⋯O11	0.75 (3)	1.91 (3)	2.568 (4)	147 (4)
O13*A*—H13*C*⋯O12	0.75 (3)	1.90 (4)	2.458 (8)	131 (4)
O33—H33*B*⋯O31	0.74 (3)	1.90 (3)	2.567 (3)	149 (4)
O33*A*—H33*C*⋯O32	0.75 (4)	1.90 (4)	2.511 (14)	138 (5)

**Table 2:** Comparison of selected structural characteristics (Å, °) of ternary Cu^II^ complexes with various axial ligands (*L* = DMSO, DMF, OEt_2_, H_2_O)

Compound	Cu⋯Cu	C—O (basal)	C—O (axial)	Cu—O—C	O—C—O	Reference
[Cu(II)_2_(naproxen)_4_(DMSO)_2_]	2.629 (1)	1.995 (4) 1.958 (4)	2.155 (5) 2.123 (5)	123.1 (4) 121.7 (4)	125.7 (5) 126.2 (5)	Dendrinou-Samara *et al.* (1990 ▸)
[Cu(II)_2_(diclofenac)_4_(DMF)_2_]	2.6265 (8)	1.981 (2) 1.953 (2)	2.122 (2)	124.7 (2) 121.2 (2)	125.5 (3)	Kovala-Demertzi *et al.* (1997 ▸)
[Cu(II)_2_(indomethacinate)_4_(DMF)_2_]	2.629 (2)	1.956 (7) 1.967 (7)	2.154 (6)	122.3 (6) 123.6 (6)	125.1 (9) 125.9 (8)	Guessous *et al.* (1998 ▸)
[Cu(II)_2_(niflumate)_4_(DMSO)_2_]	2.6272 (5)	1.952 (2) 1.968 (2)	2.152 (2)	117.2 (2) 130.5 (2)	123.8 (2) 124.1 (2)	Greenaway *et al.* (1999 ▸)
[Cu(II)_2_(3,5-DIPS)_4_(DMF)_2_]	2.6139 (9)	1.950 (2) 1.967 (2)	2.129 (2)	121.9 (2) 125.29 (2)	123.8 (3) 123.9 (3)	Morgant *et al.* (2000 ▸)
[Cu(II)_2_(3,5-DIPS)_4_(OEt)_2_]	2.613 (1)	1.948 (3) 1.957 (3)	2.230 (3)	119.7 (3) 127.0 (3)	124.0 (4) 124.1 (4)	Morgant *et al.* (2000 ▸)
[Cu(II)_2_(aspirinate)_4_(DMF)_2_]	2.6154 (4)	1.953 (1) 1.971 (1)	2.154 (1)	119.(1) 125.2 (1)	125.7 (2) 125.8 (2)	Viossat *et al.* (2003 ▸)
[Cu(II)_2_(niflumate)_4_(H_2_O)_2_]·4DMA	2.6439 (7)	1.952 (2) 1.970 (2)	2.128 (2)	120.9 (2) 127.2 (2)	123.8 (3) 124.6 (3)	Viossat *et al.* (2005 ▸)
[Cu(II)_2_(3,5-DIPS)_4_(DMSO)_2_]	2.6170 (7)	1.958 (2) 1.972 (2)	2.1226 (19)	122.04 (19) 125.71 (19)	123.3 (3) 123.3 (3)	This work

**Table 3:** Experimental details

Crystal data	
Chemical formula	[Cu_2_(C_13_H_17_O_3_)_4_(C_2_H_6_OS)_2_]
*M*_r_	1168.40
Crystal system, space group	Triclinic, *P* 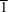
Temperature (K)	180
*a*, *b*, *c* (Å)	10.2990 (17), 11.734 (2), 12.846 (2)
α, β, γ (°)	87.275 (3), 88.918 (3), 72.096 (2)
*V* (Å^3^)	1475.6 (4)
*Z*	1
Radiation type	Mo *K*α
μ (mm^−1^)	0.85
Crystal size (mm)	0.13 × 0.08 × 0.05

Data collection	
Diffractometer	Bruker APEXII CCD
Absorption correction	Empirical (using intensity measurements) (*SADABS*; Krause *et al.*, 2015 ▸)
*T*_min_, *T*_max_	0.692, 0.746
No. of measured, independent and observed [*I* > 2σ(*I*)] reflections	19892, 6767, 4879
*R*_int_	0.046
(sin θ/λ)_max_ (Å^−1^)	0.649

Refinement	
*R*[*F* ^2^ > 2σ(*F* ^2^)], *wR*(*F* ^2^), *S*	0.047, 0.130, 1.09
No. of reflections	6767
No. of parameters	367
No. of restraints	12
H-atom treatment	H atoms treated by a mixture of independent and constrained refinement
Δρ_max_, Δρ_min_ (e Å^−3^)	0.61, −0.52

Computer programs: *APEX2* and *SAINT* (Bruker, 2014 ▸), *SHELXS97* and *SHELXTL* (Sheldrick 2008 ▸), and *SHELXL2014/7* (Sheldrick, 2015 ▸).

## supplementary crystallographic information

### Crystal data

**Table:**

[Cu_2_(C_13_H_17_O_3_)_4_(C_2_H_6_OS)_2_]	*Z* = 1
*M**_r_* = 1168.40	*F*(000) = 618
Triclinic, *P*1	*D*_x_ = 1.315 Mg m^−^^3^
*a* = 10.2990 (17) Å	Mo *K*α radiation, λ = 0.71073 Å
*b* = 11.734 (2) Å	Cell parameters from 6990 reflections
*c* = 12.846 (2) Å	θ = 2.3–28.4°
α = 87.275 (3)°	µ = 0.85 mm^−^^1^
β = 88.918 (3)°	*T* = 180 K
γ = 72.096 (2)°	Block, green
*V* = 1475.6 (4) Å^3^	0.13 × 0.08 × 0.05 mm

### Data collection

**Table:**

Bruker APEXII CCD diffractometer	4879 reflections with *I* > 2σ(*I*)
φ and ω scans	*R*_int_ = 0.046
Absorption correction: empirical (using intensity measurements) (SADABS; Krause *et al.*, 2015)	θ_max_ = 27.5°, θ_min_ = 1.6°
*T*_min_ = 0.692, *T*_max_ = 0.746	*h* = −13→13
19892 measured reflections	*k* = −15→15
6767 independent reflections	*l* = −16→16

### Refinement

**Table:**

Refinement on *F*^2^	12 restraints
Least-squares matrix: full	Hydrogen site location: mixed
*R*[*F*^2^ > 2σ(*F*^2^)] = 0.047	H atoms treated by a mixture of independent and constrained refinement
*wR*(*F*^2^) = 0.130	*w* = 1/[σ^2^(*F*_o_^2^) + (0.0587*P*)^2^ + 0.2858*P*] where *P* = (*F*_o_^2^ + 2*F*_c_^2^)/3
*S* = 1.09	(Δ/σ)_max_ = 0.001
6767 reflections	Δρ_max_ = 0.61 e Å^−^^3^
367 parameters	Δρ_min_ = −0.52 e Å^−^^3^

### Special details

**Table:**

Geometry. All esds (except the esd in the dihedral angle between two l.s. planes) are estimated using the full covariance matrix. The cell esds are taken into account individually in the estimation of esds in distances, angles and torsion angles; correlations between esds in cell parameters are only used when they are defined by crystal symmetry. An approximate (isotropic) treatment of cell esds is used for estimating esds involving l.s. planes.
Refinement. X-ray diffraction data were collected on a Bruker APEXII diffractometer using Mo-Kα radiation. The structures were solved by using direct methods and standard difference map techniques and were refined by full-matrix least-squares procedures on *F*^2^ with SHELXTL (Version 2014/7).

### Fractional atomic coordinates and isotropic or equivalent isotropic displacement parameters (Å^2^)

**Table:**

	*x*	*y*	*z*	*U*_iso_*/*U*_eq_	Occ. (<1)
Cu	0.37845 (3)	0.49460 (3)	0.47832 (2)	0.02804 (12)	
S	0.17501 (7)	0.37569 (7)	0.38084 (6)	0.0393 (2)	
O1	0.18432 (19)	0.48200 (18)	0.43872 (16)	0.0389 (5)	
C1	0.0029 (3)	0.4164 (3)	0.3402 (3)	0.0507 (9)	
H1A	−0.0116	0.3512	0.3016	0.076*	
H1B	−0.0573	0.4310	0.4014	0.076*	
H1C	−0.0178	0.4894	0.2951	0.076*	
C2	0.1689 (4)	0.2664 (4)	0.4795 (4)	0.0768 (14)	
H2A	0.1627	0.1942	0.4472	0.115*	
H2B	0.2518	0.2461	0.5218	0.115*	
H2C	0.0888	0.2984	0.5240	0.115*	
O11	0.6237 (2)	0.56862 (18)	0.37685 (15)	0.0372 (5)	
O12	0.4147 (2)	0.56118 (19)	0.34213 (15)	0.0399 (5)	
O13	0.7689 (3)	0.6204 (3)	0.2281 (3)	0.0555 (12)	0.723 (6)
H13B	0.750 (4)	0.609 (4)	0.284 (3)	0.050 (10)*	0.723 (6)
O13A	0.3219 (9)	0.6118 (9)	0.1651 (6)	0.053 (3)	0.277 (6)
H13C	0.317 (9)	0.579 (10)	0.215 (4)	0.050 (10)*	0.277 (6)
C11	0.5242 (3)	0.5824 (2)	0.3152 (2)	0.0347 (6)	
C12	0.5380 (3)	0.6236 (3)	0.2058 (2)	0.0373 (7)	
C13	0.6608 (3)	0.6379 (3)	0.1690 (2)	0.0427 (7)	
H13	0.7350	0.6248	0.2157	0.051*	0.277 (6)
C14	0.6762 (4)	0.6711 (3)	0.0650 (3)	0.0577 (10)	
C15	0.5636 (5)	0.6937 (3)	0.0008 (3)	0.0617 (10)	
H15A	0.5717	0.7184	−0.0698	0.074*	
C16	0.4389 (5)	0.6821 (3)	0.0352 (3)	0.0624 (11)	
C17	0.4288 (4)	0.6456 (3)	0.1381 (3)	0.0516 (9)	
H17	0.3455	0.6355	0.1630	0.062*	0.723 (6)
C21	0.8506 (7)	0.6300 (7)	−0.0806 (5)	0.161 (3)	
H21A	0.8511	0.5462	−0.0773	0.242*	
H21B	0.7839	0.6758	−0.1325	0.242*	
H21C	0.9415	0.6338	−0.1005	0.242*	
C22	0.8122 (5)	0.6828 (4)	0.0253 (3)	0.0814 (14)	
H22A	0.8843	0.6370	0.0758	0.098*	
C23	0.8107 (5)	0.8131 (5)	0.0221 (4)	0.0937 (16)	
H23A	0.7858	0.8459	0.0910	0.141*	
H23B	0.9015	0.8176	0.0026	0.141*	
H23C	0.7439	0.8596	−0.0294	0.141*	
C24	0.3386 (9)	0.6856 (6)	−0.1406 (5)	0.208 (5)	
H24A	0.4008	0.6039	−0.1461	0.313*	
H24B	0.2521	0.6921	−0.1746	0.313*	
H24C	0.3798	0.7427	−0.1746	0.313*	
C25	0.3147 (7)	0.7109 (6)	−0.0361 (4)	0.109 (2)	
H25A	0.2630	0.6559	−0.0095	0.131*	
C26	0.2219 (6)	0.8322 (9)	−0.0186 (5)	0.188 (4)	
H26A	0.2098	0.8436	0.0564	0.282*	
H26B	0.2610	0.8920	−0.0503	0.282*	
H26C	0.1333	0.8415	−0.0504	0.282*	
O31	0.31003 (18)	0.65648 (16)	0.53077 (16)	0.0351 (5)	
O32	0.51797 (18)	0.66681 (17)	0.56497 (15)	0.0338 (4)	
O33	0.0967 (2)	0.8246 (2)	0.5838 (2)	0.0379 (7)	0.859 (5)
H33B	0.138 (3)	0.772 (3)	0.555 (3)	0.050 (10)*	0.859 (5)
O33A	0.5527 (15)	0.8378 (14)	0.6593 (16)	0.054 (6)	0.141 (5)
H33C	0.578 (12)	0.789 (14)	0.622 (14)	0.050 (10)*	0.141 (5)
C31	0.3898 (3)	0.7105 (2)	0.5652 (2)	0.0295 (6)	
C32	0.3274 (3)	0.8303 (2)	0.6083 (2)	0.0286 (6)	
C33	0.1852 (3)	0.8809 (2)	0.6158 (2)	0.0304 (6)	
H33	0.1274	0.8388	0.5912	0.036*	0.141 (5)
C34	0.1278 (3)	0.9922 (3)	0.6591 (2)	0.0313 (6)	
C35	0.2159 (3)	1.0517 (2)	0.6925 (2)	0.0320 (6)	
H35A	0.1779	1.1284	0.7207	0.038*	
C36	0.3580 (3)	1.0038 (2)	0.6866 (2)	0.0317 (6)	
C37	0.4114 (3)	0.8930 (2)	0.6445 (2)	0.0320 (6)	
H37	0.5076	0.8585	0.6401	0.038*	0.859 (5)
C41	−0.0798 (3)	1.1751 (3)	0.6886 (3)	0.0557 (9)	
H41A	−0.1792	1.1996	0.6968	0.084*	
H41B	−0.0387	1.1936	0.7513	0.084*	
H41C	−0.0558	1.2185	0.6279	0.084*	
C42	−0.0267 (3)	1.0413 (3)	0.6728 (2)	0.0398 (7)	
H42A	−0.0692	1.0259	0.6078	0.048*	
C43	−0.0731 (3)	0.9727 (4)	0.7631 (3)	0.0625 (11)	
H43A	−0.0383	0.8864	0.7522	0.094*	
H43B	−0.0377	0.9899	0.8288	0.094*	
H43C	−0.1730	0.9978	0.7659	0.094*	
C44	0.5460 (4)	1.0940 (3)	0.6422 (3)	0.0520 (9)	
H44A	0.4937	1.1331	0.5803	0.078*	
H44B	0.5954	1.1459	0.6685	0.078*	
H44C	0.6112	1.0174	0.6236	0.078*	
C45	0.4495 (3)	1.0721 (3)	0.7256 (3)	0.0394 (7)	
H45A	0.3888	1.1524	0.7456	0.047*	
C46	0.5272 (4)	1.0117 (3)	0.8226 (3)	0.0575 (10)	
H46C	0.4628	0.9984	0.8754	0.086*	
H46D	0.5923	0.9345	0.8050	0.086*	
H46A	0.5765	1.0630	0.8499	0.086*	

### Atomic displacement parameters (Å^2^)

**Table:**

	*U*^11^	*U*^22^	*U*^33^	*U*^12^	*U*^13^	*U*^23^
Cu	0.02434 (18)	0.02916 (19)	0.0328 (2)	−0.01076 (14)	0.00134 (13)	−0.00635 (14)
S	0.0299 (4)	0.0473 (5)	0.0447 (5)	−0.0169 (3)	−0.0029 (3)	−0.0084 (4)
O1	0.0289 (10)	0.0425 (12)	0.0486 (13)	−0.0146 (9)	0.0000 (9)	−0.0120 (10)
C1	0.0322 (16)	0.071 (2)	0.054 (2)	−0.0201 (16)	−0.0063 (14)	−0.0122 (18)
C2	0.077 (3)	0.070 (3)	0.097 (3)	−0.047 (2)	−0.037 (2)	0.035 (2)
O11	0.0368 (11)	0.0413 (12)	0.0333 (11)	−0.0120 (9)	0.0022 (9)	−0.0004 (9)
O12	0.0422 (12)	0.0466 (13)	0.0357 (11)	−0.0206 (10)	−0.0020 (9)	−0.0023 (9)
O13	0.0343 (18)	0.073 (3)	0.047 (2)	−0.0008 (16)	0.0075 (15)	0.0165 (18)
O13A	0.055 (6)	0.085 (7)	0.030 (5)	−0.038 (5)	−0.010 (4)	0.010 (4)
C11	0.0403 (17)	0.0274 (15)	0.0356 (16)	−0.0086 (13)	0.0035 (13)	−0.0070 (12)
C12	0.0453 (17)	0.0332 (16)	0.0315 (16)	−0.0090 (13)	0.0031 (13)	−0.0056 (12)
C13	0.0461 (19)	0.0387 (17)	0.0379 (17)	−0.0054 (14)	0.0068 (14)	−0.0040 (14)
C14	0.066 (2)	0.050 (2)	0.048 (2)	−0.0044 (18)	0.0175 (18)	0.0020 (17)
C15	0.094 (3)	0.055 (2)	0.0348 (19)	−0.020 (2)	0.009 (2)	−0.0002 (16)
C16	0.095 (3)	0.063 (3)	0.0359 (19)	−0.034 (2)	−0.0154 (19)	0.0015 (17)
C17	0.067 (2)	0.058 (2)	0.0380 (19)	−0.0307 (19)	−0.0072 (16)	−0.0014 (16)
C21	0.177 (7)	0.184 (7)	0.138 (6)	−0.074 (6)	0.114 (5)	−0.078 (5)
C22	0.069 (3)	0.098 (4)	0.057 (3)	0.000 (2)	0.034 (2)	0.018 (2)
C23	0.070 (3)	0.135 (5)	0.087 (4)	−0.048 (3)	0.016 (3)	−0.005 (3)
C24	0.321 (12)	0.114 (5)	0.131 (6)	0.036 (6)	−0.134 (7)	−0.053 (5)
C25	0.146 (5)	0.158 (6)	0.048 (3)	−0.086 (5)	−0.049 (3)	0.028 (3)
C26	0.079 (4)	0.298 (11)	0.127 (6)	0.046 (5)	−0.056 (4)	−0.092 (6)
O31	0.0268 (10)	0.0308 (11)	0.0498 (12)	−0.0105 (8)	−0.0015 (9)	−0.0120 (9)
O32	0.0240 (10)	0.0324 (11)	0.0458 (12)	−0.0087 (8)	0.0040 (8)	−0.0119 (9)
O33	0.0240 (12)	0.0396 (15)	0.0538 (17)	−0.0135 (11)	−0.0009 (11)	−0.0140 (12)
O33A	0.029 (8)	0.032 (9)	0.107 (16)	−0.014 (7)	0.004 (8)	−0.030 (9)
C31	0.0265 (14)	0.0309 (15)	0.0328 (15)	−0.0108 (12)	0.0008 (11)	−0.0041 (12)
C32	0.0241 (13)	0.0292 (14)	0.0342 (15)	−0.0104 (11)	0.0022 (11)	−0.0038 (11)
C33	0.0257 (13)	0.0326 (15)	0.0335 (15)	−0.0098 (12)	−0.0004 (11)	−0.0012 (12)
C34	0.0243 (13)	0.0356 (16)	0.0332 (15)	−0.0083 (12)	−0.0014 (11)	0.0012 (12)
C35	0.0320 (15)	0.0265 (14)	0.0338 (15)	−0.0030 (12)	0.0001 (12)	−0.0058 (12)
C36	0.0257 (14)	0.0290 (15)	0.0396 (16)	−0.0069 (11)	−0.0016 (12)	−0.0026 (12)
C37	0.0219 (13)	0.0318 (15)	0.0411 (17)	−0.0062 (11)	0.0003 (11)	−0.0049 (12)
C41	0.0314 (17)	0.053 (2)	0.073 (3)	0.0015 (15)	0.0025 (16)	−0.0090 (18)
C42	0.0234 (14)	0.0443 (18)	0.0475 (18)	−0.0039 (13)	0.0008 (13)	−0.0045 (14)
C43	0.0325 (18)	0.071 (3)	0.077 (3)	−0.0082 (17)	0.0171 (17)	0.008 (2)
C44	0.052 (2)	0.054 (2)	0.062 (2)	−0.0333 (17)	−0.0034 (17)	0.0014 (17)
C45	0.0319 (15)	0.0307 (16)	0.058 (2)	−0.0109 (13)	−0.0031 (14)	−0.0125 (14)
C46	0.057 (2)	0.074 (3)	0.052 (2)	−0.035 (2)	−0.0099 (17)	−0.0036 (19)

### Geometric parameters (Å, º)

**Table:**

Cu—O12	1.958 (2)	C21—C22	1.518 (7)
Cu—O31	1.9595 (19)	C22—C23	1.522 (7)
Cu—O32^i^	1.9672 (19)	C24—C25	1.389 (8)
Cu—O11^i^	1.972 (2)	C25—C26	1.474 (8)
Cu—O1	2.1226 (19)	O31—C31	1.278 (3)
Cu—Cu^i^	2.6170 (7)	O32—C31	1.261 (3)
S—O1	1.511 (2)	O32—Cu^i^	1.9672 (19)
S—C1	1.770 (3)	C31—C32	1.483 (4)
S—C2	1.774 (4)	C32—C37	1.394 (4)
O11—C11	1.273 (3)	C32—C33	1.404 (4)
O11—Cu^i^	1.972 (2)	C33—C34	1.394 (4)
O12—C11	1.267 (3)	C34—C35	1.388 (4)
C11—C12	1.483 (4)	C34—C42	1.527 (4)
C12—C17	1.387 (4)	C35—C36	1.400 (4)
C12—C13	1.397 (4)	C36—C37	1.379 (4)
C13—C14	1.394 (4)	C36—C45	1.517 (4)
C14—C15	1.386 (6)	C41—C42	1.517 (4)
C14—C22	1.525 (6)	C42—C43	1.532 (4)
C15—C16	1.393 (6)	C44—C45	1.514 (4)
C16—C17	1.382 (5)	C45—C46	1.516 (4)
C16—C25	1.529 (6)		
			
O12—Cu—O31	90.21 (9)	C17—C16—C25	120.0 (4)
O12—Cu—O32^i^	89.59 (9)	C15—C16—C25	122.2 (4)
O31—Cu—O32^i^	168.77 (7)	C16—C17—C12	121.3 (4)
O12—Cu—O11^i^	168.80 (8)	C21—C22—C23	110.4 (4)
O31—Cu—O11^i^	88.12 (9)	C21—C22—C14	112.3 (5)
O32^i^—Cu—O11^i^	89.91 (8)	C23—C22—C14	111.0 (3)
O12—Cu—O1	95.71 (8)	C24—C25—C26	114.0 (5)
O31—Cu—O1	95.79 (7)	C24—C25—C16	117.3 (6)
O32^i^—Cu—O1	95.41 (8)	C26—C25—C16	110.6 (4)
O11^i^—Cu—O1	95.48 (8)	C31—O31—Cu	122.11 (17)
O12—Cu—Cu^i^	83.21 (6)	C31—O32—Cu^i^	125.63 (18)
O31—Cu—Cu^i^	85.92 (6)	O32—C31—O31	123.3 (3)
O32^i^—Cu—Cu^i^	82.91 (6)	O32—C31—C32	118.8 (2)
O11^i^—Cu—Cu^i^	85.63 (6)	O31—C31—C32	117.9 (2)
O1—Cu—Cu^i^	177.99 (6)	C37—C32—C33	119.1 (3)
O1—S—C1	104.47 (14)	C37—C32—C31	119.4 (2)
O1—S—C2	105.02 (18)	C33—C32—C31	121.4 (2)
C1—S—C2	98.30 (18)	C34—C33—C32	120.9 (2)
S—O1—Cu	119.72 (11)	C35—C34—C33	117.8 (2)
C11—O11—Cu^i^	122.04 (19)	C35—C34—C42	122.4 (3)
C11—O12—Cu	125.71 (19)	C33—C34—C42	119.8 (2)
O12—C11—O11	123.3 (3)	C34—C35—C36	122.9 (3)
O12—C11—C12	118.3 (3)	C37—C36—C35	117.8 (2)
O11—C11—C12	118.4 (3)	C37—C36—C45	121.5 (2)
C17—C12—C13	119.4 (3)	C35—C36—C45	120.7 (2)
C17—C12—C11	119.8 (3)	C36—C37—C32	121.5 (2)
C13—C12—C11	120.8 (3)	C41—C42—C34	114.4 (3)
C14—C13—C12	121.0 (3)	C41—C42—C43	110.1 (3)
C15—C14—C13	117.4 (3)	C34—C42—C43	110.0 (2)
C15—C14—C22	122.2 (3)	C44—C45—C46	110.9 (3)
C13—C14—C22	120.4 (4)	C44—C45—C36	112.5 (3)
C14—C15—C16	123.1 (3)	C46—C45—C36	112.0 (3)
C17—C16—C15	117.8 (4)		
			
C1—S—O1—Cu	−167.93 (15)	C15—C16—C25—C26	−98.5 (7)
C2—S—O1—Cu	89.15 (19)	Cu^i^—O32—C31—O31	−4.4 (4)
Cu—O12—C11—O11	−4.0 (4)	Cu^i^—O32—C31—C32	175.15 (17)
Cu—O12—C11—C12	175.03 (18)	Cu—O31—C31—O32	2.6 (4)
Cu^i^—O11—C11—O12	2.6 (4)	Cu—O31—C31—C32	−176.86 (17)
Cu^i^—O11—C11—C12	−176.39 (18)	O32—C31—C32—C37	1.9 (4)
O12—C11—C12—C17	4.4 (4)	O31—C31—C32—C37	−178.6 (3)
O11—C11—C12—C17	−176.6 (3)	O32—C31—C32—C33	−176.6 (3)
O12—C11—C12—C13	−174.1 (3)	O31—C31—C32—C33	3.0 (4)
O11—C11—C12—C13	5.0 (4)	C37—C32—C33—C34	−0.2 (4)
C17—C12—C13—C14	−1.6 (5)	C31—C32—C33—C34	178.3 (2)
C11—C12—C13—C14	176.9 (3)	C32—C33—C34—C35	1.1 (4)
C12—C13—C14—C15	2.5 (5)	C32—C33—C34—C42	−176.0 (3)
C12—C13—C14—C22	−178.3 (3)	C33—C34—C35—C36	−1.4 (4)
C13—C14—C15—C16	−1.6 (6)	C42—C34—C35—C36	175.7 (3)
C22—C14—C15—C16	179.2 (4)	C34—C35—C36—C37	0.6 (4)
C14—C15—C16—C17	−0.3 (6)	C34—C35—C36—C45	−179.2 (3)
C14—C15—C16—C25	178.2 (4)	C35—C36—C37—C32	0.4 (4)
C15—C16—C17—C12	1.3 (6)	C45—C36—C37—C32	−179.8 (3)
C25—C16—C17—C12	−177.3 (4)	C33—C32—C37—C36	−0.6 (4)
C13—C12—C17—C16	−0.4 (5)	C31—C32—C37—C36	−179.1 (3)
C11—C12—C17—C16	−178.8 (3)	C35—C34—C42—C41	20.9 (4)
C15—C14—C22—C21	−42.9 (6)	C33—C34—C42—C41	−162.2 (3)
C13—C14—C22—C21	137.9 (5)	C35—C34—C42—C43	−103.7 (3)
C15—C14—C22—C23	81.2 (5)	C33—C34—C42—C43	73.3 (4)
C13—C14—C22—C23	−98.0 (4)	C37—C36—C45—C44	57.2 (4)
C17—C16—C25—C24	−146.9 (6)	C35—C36—C45—C44	−123.1 (3)
C15—C16—C25—C24	34.6 (8)	C37—C36—C45—C46	−68.6 (4)
C17—C16—C25—C26	80.0 (6)	C35—C36—C45—C46	111.2 (3)

Symmetry code: (i) −*x*+1, −*y*+1, −*z*+1.

### Hydrogen-bond geometry (Å, º)

**Table:**

*D*—H···*A*	*D*—H	H···*A*	*D*···*A*	*D*—H···*A*
O13—H13*B*···O11	0.75 (3)	1.91 (3)	2.568 (4)	147 (4)
O13*A*—H13*C*···O12	0.75 (3)	1.90 (4)	2.458 (8)	131 (4)
O33—H33*B*···O31	0.74 (3)	1.90 (3)	2.567 (3)	149 (4)
O33*A*—H33*C*···O32	0.75 (4)	1.90 (4)	2.511 (14)	138 (5)
